# Uptake of hepatitis B vaccination among nursing students in Cameroon: a mixed-methods study

**DOI:** 10.5588/pha.26.0049

**Published:** 2026-08-24

**Authors:** R. Fandie, J.L. Ngo Likeng, S.R. Douanla, J. Noeske

**Affiliations:** 1 Ministry of Public Health, Directory of Health Promotion, Yaounde, Cameroon;; 2 School of Health Sciences (ESS), Catholic University of Central Africa (UCAC), Yaoundé, Cameroon;; 3 Ministry of Economy, Planning and Regional Development, Yaounde, Cameroon;; 4 Senior Consultant, Independent, Yaounde, Cameroon.

**Keywords:** paramedical training, HBV, vaccine uptake, sub-Saharan Africa, Cameroon

## Abstract

**SETTING:**

Three purposively identified nursing education institutions (Ayos, EITMS/GS, and the Virginia Henderson Foundation) in the Centre Region of Cameroon.

**OBJECTIVE:**

To evaluate the psychological, socio-cognitive, economic, and institutional factors associated with hepatitis B (HBV) vaccine uptake among nursing students.

**DESIGN:**

A mixed-methods cross-sectional study was conducted between June and July 2025. Quantitative data (*n* = 325) were collected via self-administered electronic surveys, supplemented by qualitative focus group discussions. Quantitative analysis was performed using SPSS. Qualitative themes were synthesised via a hybrid inductive–deductive thematic analysis, using QCAmap.

**RESULTS:**

HBV vaccination was critically low at 29.54%, with only 50.04% of those vaccinated completing the full three-dose series. Multivariate analysis identified female gender, age, advanced study level, a solid knowledge level, high perceived self-efficacy, low vaccine-related anxiety, and encouragement from the educational institution as significant predictors of uptake. Qualitative findings highlighted as primary barriers: prohibitive costs, inadequate information, weak institutional mandates, and traditional prejudices.

**CONCLUSION:**

Immunisation rates among high-risk nursing students are alarmingly insufficient. To mitigate occupational risk, public health policy should mandate HBV vaccination at school matriculation and implement subsidies to facilitate economic access together with systematic sensitisation.

According to the WHO, about 254 million individuals live with a chronic hepatitis B (HBV) infection.^1^ Nine of the 17 countries presenting 75% of the global burden of HBV are in the African Region (AFRO), mostly in sub-Saharan Africa (SSA).^2^ Cameroon, where 6.1% of the general population is HBV-infected, has one of the highest infection rates (op. cit.). HBV prevention relies on vaccination for children, and on infection and prevention and control (IPC) measures and vaccination for adults, particularly populations at risk. Since 2005, Cameroon has incorporated HBV vaccination in the Expended Programme of Immunization (EPI), but complete three-dose vaccination coverage of newborns was estimated to be under 75% in 2023.^2^

Medical personnel face an enhanced risk of occupational infections.^3,4^ This applies even more to paramedical and medical students who, during clinical internships, perform procedures involving exposure to blood and other infectious body liquids while still learning basic IPC. For more than two decades, numerous studies from SSA countries have highlighted the challenges health care workers (HCWs) face regarding HBV prevention and vaccination.^5–7^ Furthermore, several recent studies have focused on vaccination coverage among medical students,^8–10^ including one in Ghana, focussing on nursing students.^11^ Main topics are contributory risk factors, infection status, risk awareness, vaccination coverage, accessibility of vaccines, absence of a vaccination policy, and established vaccination guidelines. These studies consistently show that HBV vaccination coverage among HCWs and (para)medical students is for various reasons unsatisfactory. Assessments of infection control and prevention revealed difficulties with achieving acceptable standards in Cameroonian health facilities.^12,13^ In 2020, the Ministry of Public Health (MPH) issued a National Strategic Plan for Control of Viral Hepatitis, 2020–2024.^14^ In 2022, there followed the National Guidelines for Infection Prevention and Control in Health Facilities.^15^ Although both documents acknowledge the imperative of HBV control among health personnel, they omit to elaborate on the necessary operational intervention measures.

Three earlier, descriptive, questionnaire-based studies assessed the HBV vaccination status among medical students at Cameroonian universities.^16–18^ However, their data can be considered as outdated. There are no data on HBV vaccination among nursing students in Cameroon. Given this background, this study aimed to explore HBV vaccination coverage and the factors influencing vaccination uptake among nursing students in a convenience sample in the Centre Region of Cameroon.

## METHODS

Our cross-sectional, descriptive study employed an explanatory sequential design: quantitative data collection and analysis were followed by a qualitative phase to further contextualise and explain the statistical results.^19^ Quantitative data were collected online using semi-structured, auto-administered questionnaires via Google Forms. Qualitative data were gathered through focus group discussions (FGDs). Our study adhered to GRAMMS guidelines for mixed-methods research, with the quantitative and qualitative components following STROBE and COREQ standards, respectively.^20–22^ Reporting checklists and detailed study instruments are available via Zenodo at https://doi.org/10.5281/zenodo.20427022.

### Setting and site selection

A purposive sampling strategy was employed to select both the geographical region and the participating nursing schools. The Centre Region, particularly the capital city of Yaounde, was selected due its unique status as a national educational and professional hub. Yaounde attracts students from across the country due to its diverse training and future employment opportunities. Selecting Yaounde ensured that the study captured perspectives from students originating from various parts of the country. The three nursing schools were chosen through a purposive, maximum variation sampling approach to enhance representativeness of the findings: two schools are in the capital, and the third is in a semi-peripheral area, comprising one public and two private institutions. To capture the experiences of students from both linguistic contexts, one of the private schools is bilingual (English and French).

### Study population and participant recruitment

The study population comprised students across all three study years enrolled in any of the three selected nursing schools in the Centre Region of Cameroon: the State Registered Nurses School of Ayos, École des Infirmiers et Techniciens Médico-Sanitaires/Génie Sanitaire, and the School for the Training of Health Personnel Virginia Henderson Foundation. Data collection took place between 26th June and 15th July 2025.

A purposive, criterion-based approach was used for recruitment of participants. Eligible participants had to be full-time nursing students in their first, second, or final year at one of the three selected institutions. They had to be a minimum of 18 years of age, possess sufficient proficiency in either English or French, and given written consent to participate.

To estimate the prevalence of HBV coverage, we calculated the required sample size using G*Power 3.1, applying the formula for a single population proportion: *n* = *Z*^2^ · *p* · (1 − *p*)/*E*^2^.^23^ The expected proportion (*p*) was set at 0.30 (30% coverage), based on findings from a preliminary exploration. A 95% confidence level (corresponding to a *Z*-score of 1.96) and a margin of error (*E*) of 5% were chosen, yielding a minimum required sampling size (*n*) of 323 students (*n* = 1.96^2^ · 0.30 · 0.70/0.05^2^ ≅ 322.69). An adjustment for an estimated non-response of 20% was applied, yielding a target sample size of 404 students.

### Data collection tools

Quantitative data were collected with a questionnaire – covering domains of socio-demographic characteristics, knowledge, attitudes, behaviours, and the institutional environment – validated previously among university students enrolled in health sciences (see Supplementary Data, Section D). Class representatives in the three participating schools were identified with the assistance of the schools’ administrative staff. Upon consenting to participate in the process, the representatives were then tasked by a member of the research team to distribute anonymous questionnaires (via Google Form including a consent statement) to all students in their respective classes.

Qualitative data collection was organised as follows: After the completion of the questionnaires, participating class representatives helped to identify students who expressed interest to participate in a FGD. Data were then collected in three FGDs, one per participating school, led by a member of the study team (RF). Each discussion began with a clear explication of its objectives, the procedure for recording the session, and an oral consent declaration by each participant. A semi-structured discussion guide covering the themes was derived from the questionnaire and the study objective (see Supplementary Data Section E). The purpose was to explore shared experiences, common perceptions, and group norms, and to enrich the findings from the main quantitative sample. The sessions were audio-recorded and transcribed verbatim.

### Data processing and analysis

Questionnaires, socio-demographic information, and other quantitative responses were analysed using Microsoft Excel and IBM SPSS Statistics (v. 30.0). For the purpose of analysis, continuous scores were transformed into categorical variables. Knowledge scores were dichotomised using a criterion-based threshold (70%). Scores for risk perception, anxiety, and self-efficacy were categorised into ‘high’ and ‘low’ levels based on the sample median. Bivariate associations between socio-demographic, psychosocial, and institutional predictors and vaccination status were assessed using Pearson’s χ² test or Fisher’s exact test as appropriate. All variables demonstrating statistical significance (*P* < 0.05) were subsequently entered into a single multivariable logistic regression model to allow for identification of independent predictors while adjusting for potential confounders.

For the qualitative approach, three FGDs were held, each comprising 7–10 participants. Discussions continued until thematic saturation was reached, meaning no new insights emerged. Verbatim transcripts were managed in QCAmap software for systematic qualitative text analysis.^24^ We employed a hybrid inductive-deductive thematic analysis following Braun and Clarke’s framework.^25^ Two of the authors (RF and JN), who have experience in qualitative research, independently generated initial codes (e.g., ‘vaccination costs’ and ‘vaccination encouragement’) to develop a consensual coding framework through iterative peer debriefing. Themes were subsequently refined, defined, and supported by data extracts. Researcher reflexivity was maintained using a detailed journal to track and mitigate potential bias. Qualitative findings were ultimately integrated with the quantitative data using a joint display approach to elucidate and contextualise significant or unexpected statistical results.

### Ethical statement

The study protocol was approved by the Comité Institutionnel d’Éthique de la Recherche en Santé Humaine of the École des Sciences de la Santé de l'Université Catholique d'Afrique Centrale under number 0225/020129/CEIRSH/ESS/MSC. From each study participant, written consent was obtained prior to completing the questionnaire and participating in the FGD.

## RESULTS

A total of 330 students completed the questionnaire. Five questionnaires were excluded from the analysis because they were incomplete, resulting in a total of 325 (98.5%) valid questionnaires for analysis. The socio-demographic characteristics of the study population, together with their vaccination profile, are presented in Table 1.

**TABLE 1.** Socio-demographic and vaccination profile of the study population (N = 325).

Variables	Categories/responses	n	%
1. Socio-demographic characteristics			
Nursing school	AYOS	151	46.5%
	Virginia Henderson	100	30.8%
	EITMS/GS	74	22.8%
Sex	Female	202	62.2%
	Male	123	37.8%
Age group (median = 23 years)	≥23 years	184	56.6%
	<23 years	141	43.4%
Level of study	1st Year	77	23.7%
	2nd Year	145	44.6%
	3rd Year	103	31.7%
Marital status	Single	284	87.4%
	Married/in a union	41	12.6%
Monthly budget	≤50,000 FCFA	256	78.8%
	>50,000 FCFA	69	21.2%
2. Vaccination status			
Vaccination level	Fully vaccinated	49	15.1%
	Partially vaccinated	47	14.5%
	Uncertain	229	70.4%
Booster dose	None	325	100%
Proof of vaccination^A^	Provided (among vaccinated)	6	6.3%

A Calculated only among participants who reported being vaccinated (n = 96).

Women (62.2%), individuals aged ≥23 years (56.6%), students in their 2nd or 3rd year (76.3%), and single participants (87.4%) constitute the majority. Furthermore, students living with a monthly budget of less than 50,000 FCFA (≈88.00 USD) represent the largest economic group (78.8%). Complete vaccination coverage is 15.1%, and 14.5% are partially vaccinated, totalling 29.6%. A significant ‘vaccination literacy’ gap exists, with 70.4% of students unaware of their vaccination status. Furthermore, among those who are fully vaccinated, only 6.3% had actually verified their status.

In Table 2, we analyse how socio-demographic, cognitive, psychological, and institutional factors are associated with vaccination status. This table includes variables identified during bivariate analysis, as well as those whose significance was subsequently confirmed via multivariate logistic regression.

**TABLE 2.** Bi- and multivariable logistic regression analysis of factors associated with HBV vaccination status among nursing students (N = 325).

Variable	COR (95% CI)	*P* value	AOR (95% CI)	*P* value
Socio-demographic characteristics				
Men	1 [Ref.]	–	1 [Ref.]	–
Women	2.46 (1.44–4.21)	0.001	2.37 (1.27–4.41)	0.007
1st year of study	1 [Ref.]		1 [Ref.]	–
2nd/3rd year of study	3.42 (1.75–6.66)	<0.001	3.04 (1.33–6.91)	0.008
Monthly budget <50.000 FCFA	1 [Ref.]		1 [Ref.]	–
Monthly budget ≥50.000 FCFA	1.89 (1.08–3.29)	0.034	1.33 (0.69–2.56)	0.398 (ns)
Cognitive factors				
Knowledge index score, high (≥70%)	1 [Ref.]		1 [Ref.]	–
Knowledge index score, low (<70%)	2.16 (1.29–3.64)	0.005	2.10 (1.13–3.89)	0.019
Psychosocial factors				
Self-efficacy, low	1 [Ref.]		1 [Ref.]	–
Self-efficacy, high	2.21 (1.29–3.78)	0.005	2.10 (1.11–3.94)	0.022
Anxiety towards vaccines, high	1 [Ref.]		1 [Ref.]	–
Anxiety towards vaccines, low	3.15 (1.81–5.48)	<0.001	2.89 (1.49–5.57)	0.002
Institutional factors				
Encouragement by school: No	1 [Ref.]		1 [Ref.]	–
Encouragement by school: Yes	4.41 (2.66–7.31)	<0.001	3.92 (2.07–7.43)	<0.001
Encouragement by clinical supervisors: No	1 [Ref.]		1 [Ref.]	–
Encouragement by clinical supervisors: Yes	2.96 (1.80–4.89)	<0.001	1.81 (0.99–3.33)	0.055 (ns)

HBV = hepatitis B, AOR = adjusted odds ratio; COR = crude odds ratio; CI = confidence interval; ns = non-significant; Ref. = Reference.

Factors such as age, marital status, history of blood exposure, and the role of peers or the private social environment – as well as risk perception and confidence in health authorities – did not show significant associations during bivariate screening and, thus, we excluded them from further modelling.

While a monthly available budget of ≥50,000 FCFA and encouragement by clinical supervisors were significant in our univariate analysis, these variables did not retain statistical significance in the multivariable logistic regression model. Ultimately, we identified as robust predictors of vaccination status female gender, advanced study level (2nd and 3rd year), a solid knowledge base regarding HBV, high perceived self-efficacy, low vaccine-related anxiety, and encouragement from the educational institution. While the majority of the ‘facilitating’ factors identified here are intuitively clear, the statistical model contains some surprising findings. Most notable are female gender as well as the lack of association with the monthly available budget and the encouragement from clinical tutors in the final model. To further contextualise these results, the themes that emerged from the FGDs are presented in what follows.

The analysis of the FGDs integrates eight themes identified in Table 3. As positive drivers, participants identified systematic sensitisation and institutional obligation (Themes T1–T2). Critical barriers, however, constituted for them the deficit on information and sensitisation, the cost of the vaccine, the failure of institutional and personal accountability, social–cultural influences, and, less prevalent, the fear of side-effects (Themes T3–T7).

**TABLE 3.** Themes (T) identified during focus group discussions.^A^

	Quote
Facilitating factors	
T1: Systematic sensitisation	‘Everything depends on the establishment…the teachers can transfer their knowledge. And like that one can adapt this (i.e., the necessity to get vaccinated – RF) with one’s emotions.’ (P10-FGD2)^B^
T2: Institutional obligation	‘…, according to me, I would say that vaccination, in particular against the HBV, normally should be an obligation in every establishment.’ (P9-FGD2) ‘… there are certain clinics, if you arrive they are imposing HBV vaccination before starting the clinical placement. If you don’t get vaccinated you are not admitted. There it’s becomes automatic (i.e., to get vaccinated – RF).’ (P8-FGD2)
Critical barriers	
T3: Deficit of information in the school	‘… to participate (at the HBV vaccination – RF) in our establishment – almost nobody is talking about…, hepatitis is not vulgarised and even sensitisation is not done.’ (P1-FDG3) ‘There is no information…’ (P2-FGD1) ‘…sensitisation sessions have to be done….’ (P4-FDG2)
T4: Cost of vaccination	‘First, the price is a problem…It’s expensive, the vaccines are expensive.’ (P5-FGD1) ‘…one has to reduce the price of the vaccine.’ (P2-FGD1)
T5: Failure of institutional and pedagogical accountability	‘…Government, facing a contagious pathology, should intervene with regard to the costs of the vaccine, it should be either free-of-charge or subsidised….’ (P5-FGD2) ‘…primarily they (i.e., health personnel in clinical placement settings – RF) have this bad way of answering our questions and not telling everything we need to know. Their answers are vague. It’s like that. Me, having lived these situations, now, if a health worker confronts me for telling me something I don’t trust him.’ (P7-FGD2)
T6: Socio-cultural influences	‘There is this problem, too, we have a certain way of thinking, we, Africans, saying that “No, it’s not good, if you’re taking it (i.e., the vaccine. – RF) you’ll die. Consequently, it is somehow difficult. Our traditional myth (!) is impacting our professional decision.”’ (P6-FGD2) ‘(Mostly we think that)… as during the vaccination campaigns only children are concerned’. (P2-FGD2).
T7: Fear of side-effects	‘… HBV vaccines as they are sub-catalysed (!), you take it and after one week you find yourself paralysed. This makes, sometimes, that you remain as you are, telling yourself: No, better yet I leave....’ (P5-FGD2)

HBV = hepatitis B; FGDs = focus group discussion.

A A total of 26 individuals participated in the three FGDs: range of age 20–29 and a total of 19 female and 7 male students.

B P10-FGD2 = Participant 10 – Focus Group Discussion 2 (French–English translations by co-author JN).

Qualitative findings added nuance to the identified quantitative findings. Two key drivers of vaccination – adequate sensitisation and knowledge alongside institutional encouragement (T1 and T2) – mirrored their corresponding barriers (i.e., the lack thereof). Participants even suggested mandating vaccination as a requirement prior to clinical placement (T2). The importance of self-efficacy is highlighted in T1: a participant declared that teachers can play a key role in reinforcing the ‘emotional impetus’ needed for vaccination. However, the most important barrier – besides lack of sensitisation – was cost (T4). Participants assigned primary responsibility to the Government, specifically the MPH, for both accountability and bearing costs (T5). The FGDs also elucidated why the score of tutor encouragement remained poor and non-significant in the final model. As one participant noted, tutors are often distrusted due to perceived ‘authoritarian’ attitudes and a perceived lack of genuine concern (T5). A noteworthy emerging theme involves traditional convictions acting as barriers, specifically ancestral beliefs and popular misconceptions about vaccination (T6). Furthermore, the anxiety identified in the quantitative data re-emerged qualitatively as a specific ‘fear of side-effects’ (T7).

## DISCUSSION

To the best of our knowledge, this study is the first to explore HBV vaccination status and its associated factors among nursing students in Cameroon. The most striking finding is the low anti-HBV vaccination coverage within this at-risk population; only 15.1% of students are fully vaccinated and 14.5% partially vaccinated, bringing the total to a mere 29.6%. Equally notable is that approximately 70% of participants were uncertain about their vaccination status. We categorised these ‘uncertain’ respondents as unvaccinated because most were born prior to the 2005 integration of the HBV vaccine into Cameroon’s EPI, and national coverage estimates remained below 75% as recently as 2023,^2^ rendering true background immunity highly unlikely in the absence of documented records.

Our findings of a 15.1% full HBV vaccination rate align with data reported by Aroke et al.,^16^ who observed that only 16.8% of Cameroonian medical students were fully vaccinated and 9.2% were partially vaccinated. Moreover, broader meta-analyses indicate that Central Africa exhibits the lowest HBV vaccination coverage in SSA, falling below 20% for medical students.^26^

Vaccination decisions are extraordinary complex. Our study opted not to utilise the recently developed 5C scale for measuring the ‘psychological antecedents of vaccination’,^27^ prioritising instead an exploratory investigation of the specific empirical context. In our study population, the identified factors ranged from psychological and socio-cultural to economic and institutional – representing a combination of conflicting drivers and structural barriers. These results correlate with findings from earlier studies among medical and nursing students, as well as recent data regarding HCWs in Cameroon.^16–18,28–30^ On the subjective level, the factors associated with the vaccination decision include a specific psychological disposition, accumulated life and academic experience, relevant knowledge regarding HBV, risk perception, and an awareness of traditional prejudices. On the structural level, key correlates include targeted institutional interventions during training, vaccine availability, and the mitigation of economic barriers by the MPH. The fact that knowledge alone does not automatically correlate with uptake is evidenced by the contradictory demand from FGD participants for mandatory vaccination, despite their inherent recognition of its necessity.^31^ The lack of statistical association between a student’s monthly disposable income and vaccination status appears less paradoxical considering that an estimated 40% of the Cameroonian population lives under the poverty line^32^; thus, even ‘higher’ income brackets remain earmarked for more essential living expenses than preventive health care.

No conclusive explanation was found for the significantly higher vaccination coverage among women. While a recent meta-analysis^26^ showed no clear gender-vaccination association, a Ugandan study^33^ linked male gender positively with screening. Our findings warrant further investigation in future studies.

The two major limitations of our study are possible social desirability bias in questionnaire responses and selection bias due to purposive sampling; thus, results should be extrapolated to the target population with caution. However, this is mitigated by the fact that our findings align with data from other regions in Cameroon and similar SSA settings.

## CONCLUSION

Uptake of HBV vaccine among Cameroonian nursing students is insufficient, strongly linked to socio-economic and institutional barriers. To mitigate occupational risk, public health policy should mandate HBV vaccination at school matriculation and implement subsidies to facilitate economic access together with systematic sensitisation.
